# Tidal marsh restoration enhances sediment accretion and carbon accumulation in the Stillaguamish River estuary, Washington

**DOI:** 10.1371/journal.pone.0257244

**Published:** 2021-09-10

**Authors:** Katrina L. Poppe, John M. Rybczyk

**Affiliations:** Department of Environmental Sciences, Western Washington University, Bellingham, Washington, United States of America; Centro de Investigacion Cientifica y de Educacion Superior de Ensenada, MEXICO

## Abstract

Tidal marshes have been recognized globally for their ability to sequester “blue carbon” but there is still a need for studies investigating the marsh response to restoration, particularly in the Pacific Northwest United States. Here we report carbon stocks and accumulation rates for restored and natural tidal marshes in the Stillaguamish River estuary in Puget Sound, Washington, where a 60-hectare marsh was reintroduced to the tidal regime from its previous use as diked and drained farmland. We found that the restoration not only maximized carbon accumulation but also enhanced resilience to rising sea levels. Four years after restoration, mean sediment carbon stocks in the upper 30 cm within the restored marsh (4.43 kg C m^-2^) were slightly lower than those measured in the adjacent natural marshes (5.95 kg C m^-2^). Mean carbon accumulation rates, however, were nearly twice as high in the restored marsh (230.49 g C m^-2^ yr^-1^) compared to the natural marshes (123.00 g C m^-2^ yr^-1^) due to high rates of accretion in the restored marsh (1.57 cm yr^-1^). Mean elevation change rates were nearly twice that of corresponding ^210^Pb accretion rates, but all were greater than the current rate of sea level rise.

## Introduction

Coastal wetlands are among the most valuable ecosystems in terms of the ecosystem services they provide [1] (Costanza et al. 2014) and they have been recognized for their role in blue carbon sequestration and climate change mitigation [2]. “Blue carbon” refers to the fraction of atmospheric carbon captured by the world’s oceans, typically stored in the sediments of saline coastal wetlands such as mangroves, salt marshes, and seagrasses [3]. Draining and converting coastal wetlands to other land uses often results in the release of that stored carbon back into the atmosphere [4, 5]. Restoring these same wetlands can potentially reverse that loss.

Approximately 85% of historical tidal wetlands have been lost from the west coast of the United States [6]. Restoration efforts have increased in recent decades, often with the primary goal of regaining fish and wildlife habitat, but other ecosystem benefits such as carbon sequestration have more recently been added to the list of reasons. Tidal marsh restoration can be costly however, averaging $68,000 per hectare in developed countries [7]. If the carbon sequestration benefit of restoration is quantified this could potentially open up access to new sources of restoration funding through the voluntary carbon market [8], as well as provide data to inform national greenhouse gas inventorying [9].

In the Pacific Northwest United States (PNW), neither land managers nor policy makers have had sufficient region- or site-specific quantitative carbon data needed to incorporate the value of carbon sequestration into improved coastal resource management plans or to support management actions through carbon finance mechanisms. Although PNW blue carbon studies have increased in very recent years, few have studied carbon accumulation rates in recently restored marshes across this large and diverse region [see 10–13]; fewer still are those able to provide a measure of variability within an estuary to help us understand the replication efforts required to accurately represent an estuary. Our study provides estimates of long-term accretion and carbon accumulation rates from several sites both in a recently restored tidal marsh and adjacent natural tidal marshes of the Stillaguamish estuary in Port Susan Bay, Puget Sound, Washington. We also evaluated relationships between environmental variables (elevation and salinity) and plant biomass, sediment carbon stocks, and carbon accumulation rates.

## Methods

### Study area

**Fig 1:**
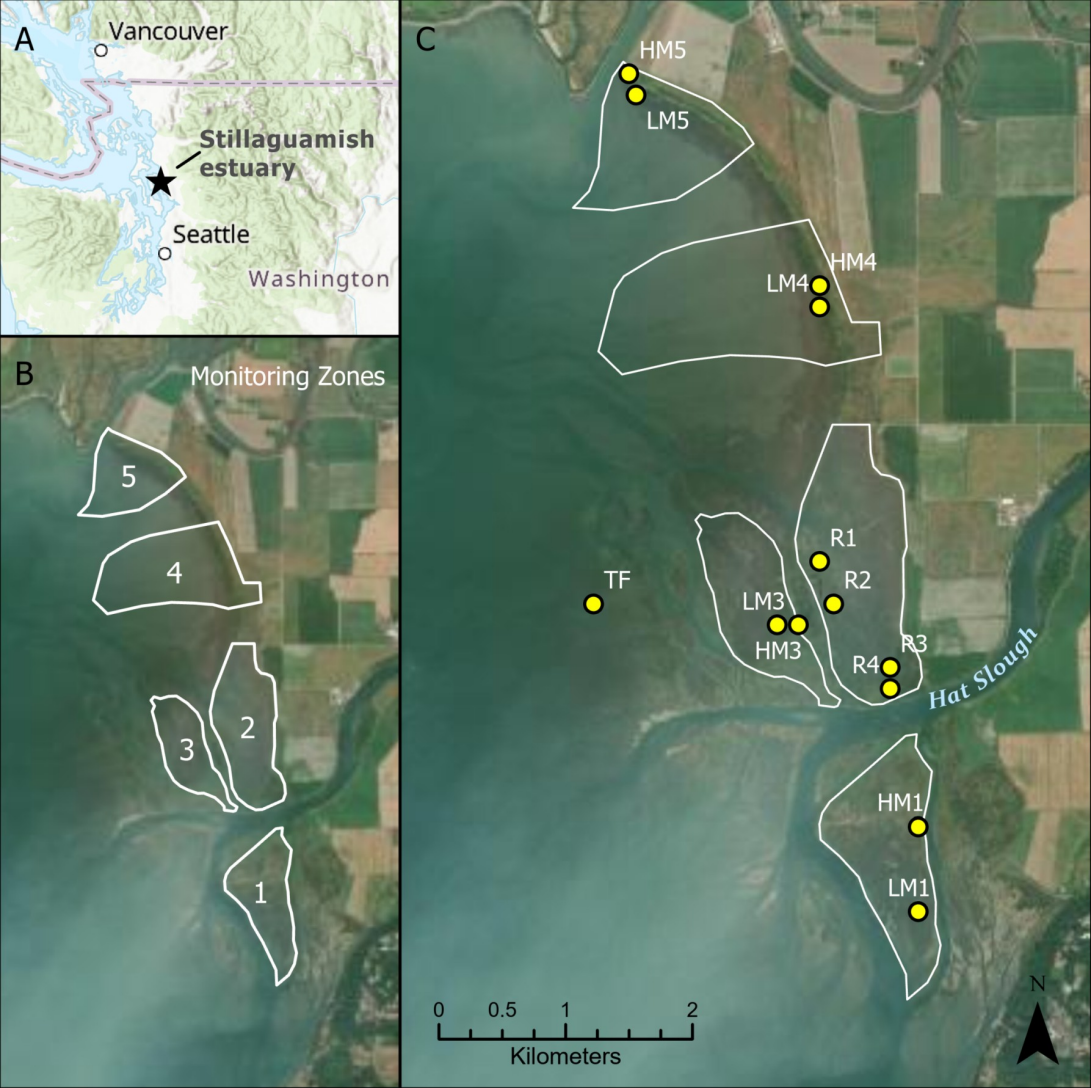
Location of (A) Stillaguamish estuary within Washington state, USA, (B) monitoring zones within the estuary, and (C) study sites for this study. Study sites are denoted with yellow circles (HM = high marsh, LM = low marsh, R = restored, TF = tidal flat). Zone 2 is the 60-hectare restored marsh. ArcGIS World Imagery basemap source: Esri, Maxar, GeoEye, Earthstar Geographics, CNES/Airbus DS, USDA, USGS, AeroGRID, IGN, and the GIS user community.

The Stillaguamish River discharges into the Port Susan Bay Preserve, owned by The Nature Conservancy. The Stillaguamish River drains a 1800 km^2^ watershed largely fed by rain and snowmelt from the North Cascade Range and the Puget lowlands. This mesotidal estuary (2.3 m mean tidal range) faces south-southwest, almost directly facing the direction of predominant winter storm winds, resulting in a storm wave fetch of up to 35–50 km. The nearest tide station provides a local rate of relative sea level rise of 1.89 mm/yr over the period 1972–2019 [14]. The Port Susan Bay Preserve contains a 60-hectare marsh restoration site that was reintroduced to tide and river pulses via dike lowering and two dike breaches in 2012. Prior to the restoration, the site had been managed as diked and drained farmland since the 1950s, and during this period it had subsided up to 1 m in elevation. Since 2011, the wetlands research group at Western Washington University (WWU) has partnered with The Nature Conservancy to conduct pre- and post-restoration monitoring both inside and outside the restoration area (Fig 1).

Beginning in 2011, we established study sites in five monitoring zones (Fig 1). This included the establishment of 21 permanent surface elevation tables (SETs) [15] to measure long-term elevation change throughout the estuary. These zones represent a range of conditions from a healthy, undisturbed marsh (Zone 1), to a regenerating, rapidly accreting emergent marsh in the restoration zone (Zone 2). The study zones also vary hydrologically, with some located directly adjacent to the Stillaguamish River mouth (shown as Hat Slough) and others more distant from the river and its active distributaries (e.g., Zones 4 and 5).

Except for the restoration zone, each of the zones consist of two sites (high and low marsh relative to MHW) and each site has two SETs. The restoration zone consists of four low marsh sites each containing one SET; there is currently no high marsh there due to the 0.5–1.0 m of subsidence prior to restoration. An additional site is located in the lower-elevation tidal flats for a total of 13 sites.

### Field sampling

Field sampling permission was provided by The Nature Conservancy as the owner of our entire study area. In the spring and summer of 2016, at each of our 13 sites, we collected two replicate cores to determine soil carbon stocks and vertical accretion rates. At sites containing two established SETs, each replicate core was paired with a SET and located randomly within 10 m of that SET. At sites containing only one SET, replicate cores were both located randomly within 10 m of the same SET. PVC coring tubes (10.2 cm internal diameter) were driven up to one meter into the sediment with a sledgehammer. Cores were kept upright during transport to the laboratory and frozen for later processing.

Aboveground and belowground plant biomass samples were collected at the end of the 2016 growing season (late August) from three replicate plots (0.15 m^2^) distributed randomly at each site. All aboveground biomass within each plot was clipped to the sediment surface. Belowground biomass samples were collected by pushing PVC coring tubes (10.0-cm internal diameter) to a depth of 50 cm in the center of each replicate biomass plot to capture the entire root zone. Belowground biomass cores were extracted in the field and sectioned into 10-cm depth sections. Biomass samples were stored in plastic bags for transport to the laboratory and temporary storage at 2˚C.

We measured elevation change at the 21 previously established SETs in the spring of each year through 2018, to continue building a time series of elevation change that began in 2011. Field measurement methods followed those described by Lynch et al. [15] for deep RSETs. Elevation change rates were determined using an ordinary least squares linear regression, with time as the independent variable, elevation relative to initial as the dependent variable, and the slope of the regression indicating the rate of surface elevation change. Elevation was previously measured at each SET in 2012 by the US Geological Survey (USGS) using Real Time Kinematic Global Positioning System (RTK-GPS). For this study, where sites included a pair of SETs, we averaged the two SET elevations to report one elevation for each site.

Sediment porewater salinity was measured in late summer of 2016 at the time of plant biomass collection. At each site, one of the pits remaining from the belowground biomass cores was left open to allow porewater to collect. Porewater salinity was measured in the field with a portable refractometer. A few sites were completely dry during sampling, preventing salinity measurements at those sites.

### Laboratory analyses

Plant aboveground biomass samples were washed with a 0.5-mm sieve and separated by species. Belowground biomass samples were washed and separated into live and dead root fractions, with the dead fraction then discarded to isolate end-of-season live biomass. Dead roots present in various states of decay and are thus considered a component of the soil fraction. All samples were oven dried at 60 ˚C for at least 96 hours and weighed. We used a 1:0.38 ratio to convert biomass dry weight to biomass carbon [16].

In the laboratory, frozen sediment cores were extracted, sliced into 2-cm sections with a power saw, and dried at 60 ˚C for at least 96 hours to determine bulk density. A subsample of each section was pulverized using a Wiley Mill with 0.425-mm mesh screen. The organic matter (OM) content of each section was determined by loss on ignition (LOI). Subsamples were burned at 500 ˚C for approximately 8 hours and weighed before and after burning [17].

We measured percent organic carbon (C_org_) content for a subset of samples (*n* = 124) with a FlashEA 1112 CN analyzer (Thermo Fisher Scientific, Waltham, MA). We first measured total (organic and inorganic) carbon content, then measured inorganic carbon by analyzing the ashed subsamples that remained after LOI, and finally calculated the C_org_ content by subtraction. We developed an OM-C_org_ conversion from these 124 samples that was then applied to all sediment samples to produce the C_org_ contents reported here (C_org_ = 0.0035*OM^2^ + 0.4135*OM– 0.4496; *R*^*2*^ = 0.97; *P* < 0.001) (S1 Fig). We used C_org_ for all further calculations of carbon values (carbon density, carbon stocks, carbon accumulation rates). Carbon density was calculated for each core section as the product of C_org_ content and bulk density. Each sediment characteristic parameter was averaged across the top 30 cm of each core for reporting purposes. Sediment carbon stocks were also calculated to a depth of 30 cm. However, a thick layer of organic material (presumably from a depositional event or burial of pre-restoration biomass) was observed in the restoration site cores between 10 cm and 20 cm in depth, which biased those results high when averaged across the top 30 cm. We therefore used only the top 10 cm of the restoration site profiles and extrapolated to 30 cm to best represent post-restoration conditions without the influence of this depositional layer.

We analyzed sediment grain size composition by separating fine from coarse sediments to evaluate the relationship of grain size with sediment carbon stocks. A subset of six samples distributed in even depth intervals was selected for analysis from each core. Each 30-mL subsample was redried, weighed, soaked, and rinsed through a 63-μm sieve to remove the fine sediment (silt and clay) portion. The remaining coarse-grained (sand) portion was dried and weighed.

Long-term sediment accretion rates were determined from the downcore distribution of excess ^210^Pb activity from one core per site. Our analysis was limited to one core per site due to time constraints (the analysis can require up to one month per core). We used the Canberra Germanium Detector (model GL2820R, Mirion Technologies (Canberra) Inc., Meriden, CT), with gamma emissions at 46 keV and 351 keV recorded by Genie 2000 software (Canberra 2002). Excess (unsupported) ^210^Pb was calculated as the difference between total ^210^Pb (at 46 keV) and supported ^210^Pb (at 351 keV) to distinguish between excess ^210^Pb deposited at the sediment surface and supported ^210^Pb that has decayed from radium in the sediment. Within each core, a subset of 2-cm sections was analyzed from the surface to the depth at which excess ^210^Pb declined to zero. We used the constant initial concentration (CIC) model [18] because in many cases we could not recover the full profile of excess ^210^Pb due to core length or time constraints. With CIC, a linear regression of the natural log of excess ^210^Pb activity versus depth was used to determine the sediment accretion rate, which is equal to –*λ/s*, where *λ* is the half-life of ^210^Pb (22.2 yr^-1^) and *s* is the slope of the regression. Because ^210^Pb profiles from the four restored sites showed evidence of disturbance, preventing us from assigning them a long-term accretion rate that could be compared with rates from the undisturbed sites, we used the mean ratio of ^210^Pb-based rate to SET-based rate from the eight natural marsh sites to estimate the ^210^Pb-based accretion rate for the restoration sites.

Carbon accumulation rates were calculated in two ways: 1) as the product of sediment carbon density and the ^210^Pb -based accretion rate, and 2) as the product of carbon density and the SET-based elevation change rate. These two rates are reported as the ^210^Pb-based carbon accumulation rate and the SET-based carbon accumulation rate, respectively. Although SET-based carbon accumulation rates are not as commonly reported in blue carbon studies, we hypothesized that, in concept, these rates should be comparable to ^210^Pb -based accumulation rates. We include them to 1) provide a validation for the ^210^Pb -based rates, 2) test the feasibility of using SET and ^210^Pb accretion rates interchangeably, and 3) provide a potential alternative to the ^210^Pb -based rates in the event that any cores were unable to be dated with ^210^Pb due to sediment mixing or other disturbance.

Statistical analyses were conducted in the R programming environment using a critical value (α) of 0.05 [19]. We used the Shapiro-Wilk test to check for normality of biomass and core data. We used simple linear regression to determine the relationship between organic content and C_org_ content. We used Spearman’s rank correlation to evaluate relationships between environmental variables and plant biomass, sediment carbon stocks, and carbon accumulation rates, and Kruskal-Wallis H test to compare high and low marsh biomass.

## Results

### Plant biomass and biomass C stocks

**Fig 2:**
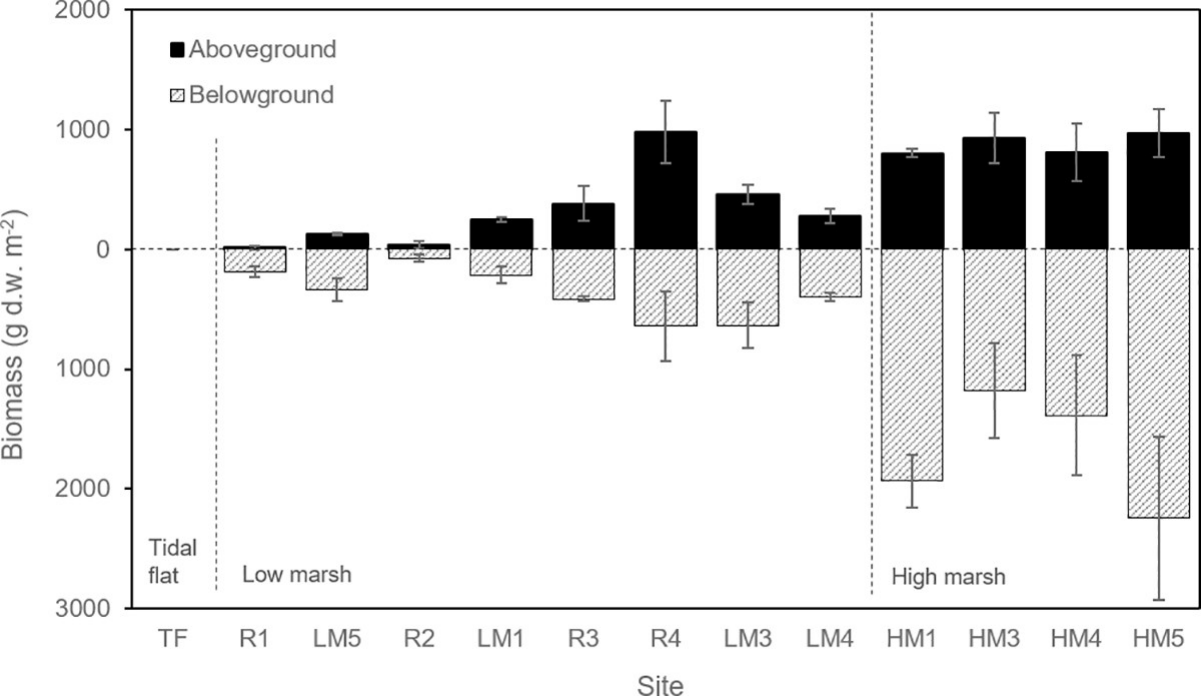
Aboveground and belowground biomass by site. Sites are arranged in order of increasing elevation. The elevations of the restored sites (R1 –R4) were within the range of other low marsh sites. Error bars represent standard error.

Aboveground biomass ranged from 0 g dw m^-2^ (at Site TF, the tideflat site) to 978.64 g dw m^-2^ (HM5) (Fig 2). Aboveground biomass at low marsh sites averaged 279.09 g dw m^-2^, compared to 879.40 g dw m^-2^ at high marsh sites, with 353.37 g dw m^-2^ across the four restoration area sites. Belowground biomass across all sites was an average of 2.4 times greater than aboveground biomass (median of 1.6), ranging from 0 g dw m^-2^ (TF) to 2,243.98 g dw m^-2^ (HM5). Belowground biomass at low marsh sites averaged 397.83 g dw m^-2^, compared to 1,686.54 g dw m^-2^ at high marsh sites, with 329.11 g dw m^-2^ at restoration area sites.

### Sediment characteristics

The mean sediment bulk density observed at both natural and restored marsh sites was identical, at 0.92 g cm^-3^, lower than the tidal flat bulk density of 1.48 g cm^-3^ (Table 1). In general however, organic matter content, organic carbon content, and carbon density values were highest at natural marsh sites, intermediate at restored sites, and lowest at the tidal flat site. Organic matter content averaged 6.45% at natural marsh sites, 4.93% at restored sites, and 2.70% at the tidal flat site. Carbon content averaged 2.38% at natural marsh sites, 1.74% at restored sites, and 0.70% at the tidal flat site. Carbon density averaged 0.020 g C cm^-3^, 0.015 g C cm^-3^, and 0.010 g C cm^-3^, respectively.

**Table 1:** Site elevation, marsh type, porewater salinity, and sediment characteristics. Sediment characteristics (bulk density, organic content, carbon content, carbon density, and grain size) are reported as the average value across the top 30 cm. Elevation is given relative to mean lower low water (MLLW).

	Site	Marsh type	Elevation (m MLLW)	Porewater salinity (ppt)	Bulk density (g cm^-3^)	Organic content (% by weight)	Carbon content (% by weight)	Carbon density (g C cm^-3^)	Grain size (% fines)
*Natural marsh*			* *						
	HM5	High	3.34	−	0.92	7.60	2.90	0.026	85.77
	LM5	Low	2.48	20	1.19	4.39	1.44	0.016	66.65
	HM1	High	3.04	6	0.82	7.18	2.73	0.020	68.50
	LM1	Low	2.65	12	1.36	3.26	0.94	0.012	42.88
	HM3	High	3.24	20	0.84	6.70	2.49	0.020	81.62
	LM3	Low	2.86	20	0.76	7.11	2.67	0.020	67.10
	HM4	High	3.27	−	0.76	7.86	3.02	0.023	98.60
	LM4	Low	2.86	15	0.72	7.49	2.85	0.020	97.06
		**Mean**	**2.97**	**15.5**	**0.92**	**6.45**	**2.38**	**0.020**	**76.02**
		SE	0.11	2.3	0.08	0.60	0.27	0.00	6.55
*Unveg tideflat*									
	TF		**2.15**	**−**	**1.48**	**2.70**	**0.70**	**0.010**	**23.46**
*Restored marsh*									
	R1	Low	2.40	17	1.10	3.99	1.26	0.013	78.22
	R2	Low	2.50	19	0.83	5.56	1.96	0.016	83.90
	R3	Low	2.76	15	0.82	5.57	2.20	0.016	73.44
	R4	Low	2.85	11	0.92	4.60	1.53	0.014	71.08
		**Mean**	**2.62**	**15.5**	**0.92**	**4.93**	**1.74**	**0.015**	**76.66**
		SE	0.11	1.7	0.06	0.39	0.21	0.001	2.83

The percentage of fine-grained sediments, as averaged over the top 30 cm, was lowest (23.46%) at the tidal flat site (TF), and highest (98.60%) at high marsh site HM4. Although the percentage of fines was more variable across the natural marsh sites, the mean from both natural marsh sites and restored sites was approximately equal, with 76.02% and 76.66% fines, respectively (Table 1). As expected, sediment grain size from individual samples was significantly correlated with sample organic content (Spearman’s rank correlation, ρ = 0.78, *P* < 0.001, *n* = 151). However, a visual assessment of the relationship suggests that the percentage of fine-grained sediments best correlated with the minimum organic content rather than the full range of observed values at any given site (S2 Fig).

### Elevation change rates

**Fig 3:**
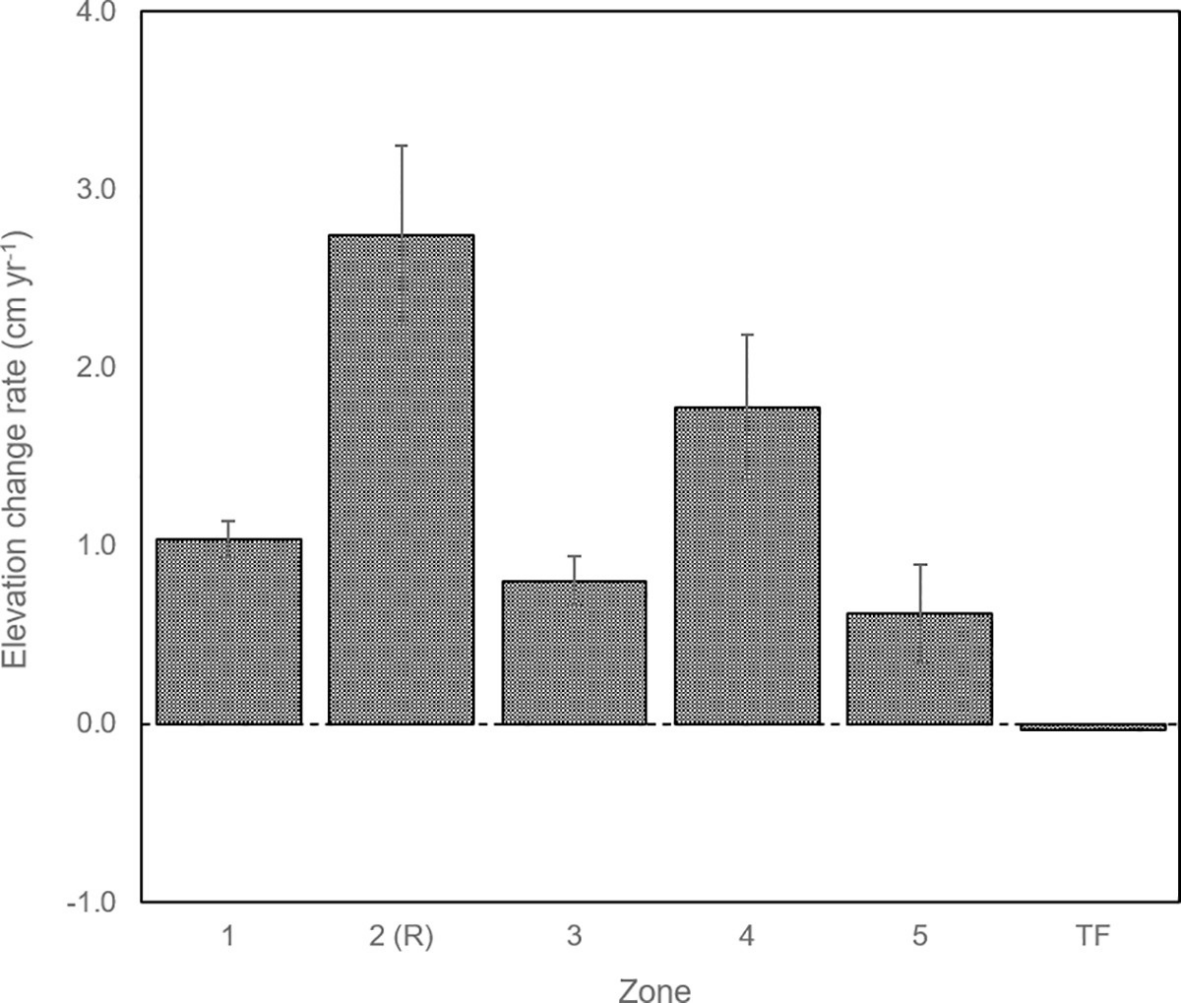
Mean rate of elevation change as measured by SETs and averaged by zone, for the entire period of record (2011–2018) with standard error bars. Zone 2 is the restored area, Zone TF is the tidal flat zone, and the remaining Zones 1, 3, 4, and 5 are natural marsh zones (see Fig 1 for a map showing the location of all zones).

Rates of surface elevation change measured with SETs ranged from -0.03 cm yr^-1^ (TF) to 3.79 cm yr^-1^ (R2) (Table 2). The mean rate across the eight natural marsh sites was 1.03 cm yr^-1^ (Fig 3), while the mean rate across the four restored sites was nearly three times higher at 2.74 cm yr^-1^.

**Table 2:** Rates of elevation change, accretion, and carbon accumulation, and carbon stocks across the three carbon pools. SET elevation change rates represent a 4–7 year time period of record, while ^210^Pb accretion rates represent an approximately 100-year record. Both C accumulation rates incorporate the 30-cm average soil carbon density.

	Site	SET elevation change rate	^210^Pb accretion rate	SET-based	^210^Pb-based	Sediment C stock in top 30 cm	Aboveground biomass C stock	Belowground biomass C stock
				C accumulation rate	C accumulation rate			
		(cm yr^-1^)	(cm yr^-1^)	(g C m^-2^ yr^-1^)	(g C m^-2^ yr^-1^)	(kg C m^-2^)	(kg C m^-2^)	(kg C m^-2^)
*Natural marsh*								
	HM5	0.67	0.34	177.53	90.09	7.95	0.37	0.85
	LM5	0.38	0.24	61.63	39.45	4.93	0.05	0.13
	HM1	0.88	0.28	176.81	56.26	6.03	0.31	0.74
	LM1	1.20	0.30	148.63	37.31	3.73	0.09	0.08
	HM3	0.73	0.60	147.88	121.55	6.08	0.35	0.45
	LM3	0.84	0.82	166.79	162.82	5.96	0.17	0.24
	HM4	2.46	1.24	561.02	282.79	6.84	0.31	0.53
	LM4	1.09	0.95	222.28	193.73	6.12	0.11	0.15
	**Mean**	**1.03**	**0.60**	**207.82**	**123.00**	**5.95**	**0.22**	**0.40**
	SE	0.22	0.13	52.95	30.47	0.44	0.05	0.10
*Unveg tideflat*								
	TF	**-0.03**	**0.00**	**-2.95**	**0.00**	**2.95**	**0.00**	**0.00**
*Restored marsh*								
	R1	2.98	1.71*	394.24	226.22	3.97	0.01	0.07
	R2	3.79	2.17*	611.69	350.23	4.84	0.01	0.03
	R3	1.37	0.78*	217.85	124.03	4.77	0.15	0.16
	R4	2.82	1.61*	387.95	221.49	4.13	0.37	0.24
	**Mean**	**2.74**	**1.57**	**402.93**	**230.49**	**4.43**	**0.13**	**0.13**
	SE	0.50	0.29	80.69	46.34	0.22	0.09	0.05

* ^210^Pb accretion rates at restored marsh sites were estimated with a ^210^Pb:SET rate conversion (see Methods section for further details).

### Long-term accretion rates

Long-term accretion rates determined from ^210^Pb dating ranged from 0 cm yr^-1^ (TF) to 2.17 cm yr^-1^ (R2) (Table 2). The mean accretion rate at natural marsh sites was 0.60 cm yr^-1^, while the mean rate at restored sites was 1.57 cm yr^-1^. The restoration site ^210^Pb profiles appeared disturbed, with discontinuous layers and a mixed surface layer corresponding to the most recent post-restoration period, therefore the mean ^210^Pb:SET ratio of 0.57 was applied to SET-based rates at these four sites to estimate the ^210^Pb accretion rate. ^210^Pb accretion rates varied more among zones than within zones, although a Kruskal-Wallis test determined this pattern to be not significant (*H* = 10.14, 5 *df*, *P* = 0.07).

Most cores demonstrated anomalous ^137^Cs profiles with low activities and no obvious peak, indicating either post-depositional vertical mobility [20] or peak deterioration due to radionuclide decay [21], therefore we do not report any ^137^Cs-derived accretion rates.

### Carbon stocks and accumulation rates

**Fig 4:**
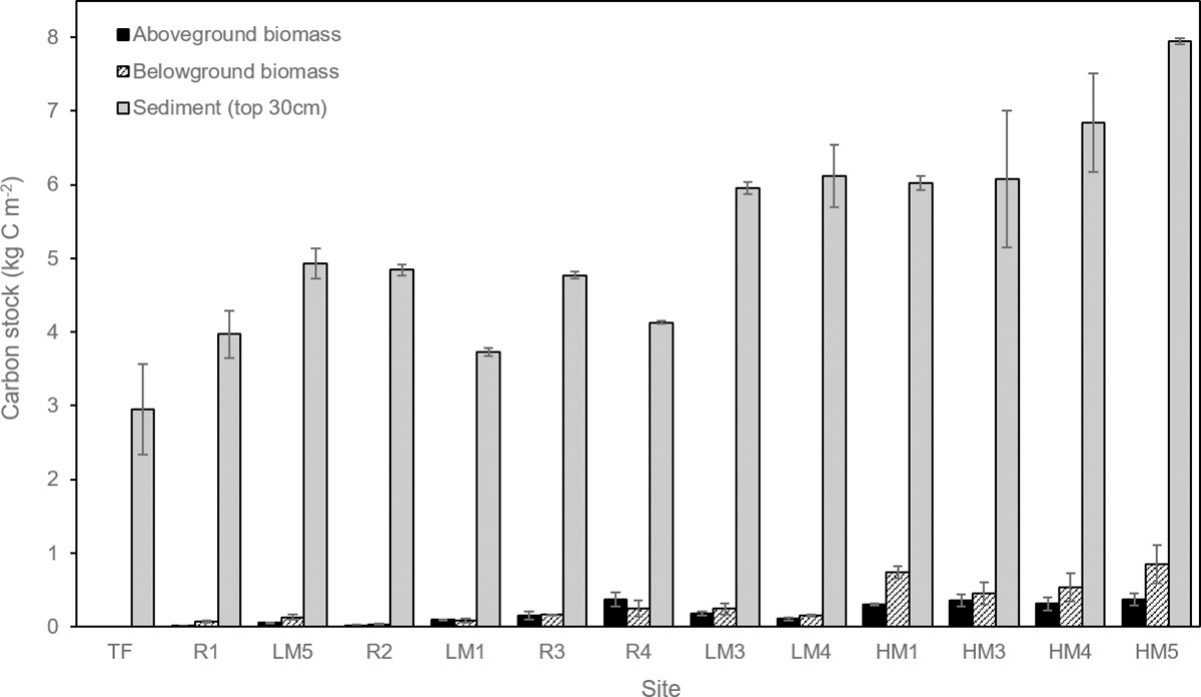
Carbon stocks (±SE) in aboveground biomass, belowground biomass, and the top 30 cm of sediment at each site. Sites are arranged in order of increasing elevation.

Sediment carbon stocks in the top 30 cm ranged from 2.95 kg C m^-2^ (TF) to 7.95 kg C m^-2^ (HM5) (Table 2). As with other sediment characteristics, restored site carbon stocks were intermediate between the tidal flat site and the natural marsh sites, averaging 4.43 kg C m^-2^ in restored sites, 2.95 kg C m^-2^ in the tidal flat, and 5.95 kg C m^-2^ in natural marshes. Sediment carbon stocks were approximately 30 times greater on average than biomass carbon stocks (Fig 4, Table 2). Aboveground biomass carbon stocks ranged from 0 kg C m^-2^ (TF) to 0.37 kg C m^-2^ (HM5 and R4), averaging 0.22 kg C m^-2^ at the natural marsh sites, and 0.13 kg C m^-2^ at the restored sites. Belowground biomass carbon stocks ranged from 0 kg C m^-2^ (TF) to 0.85 kg C m^-2^ (HM5), averaging 0.40 kg C m^-2^ at natural marsh sites and 0.13 kg C m^-2^ at restored sites.

**Fig 5:**
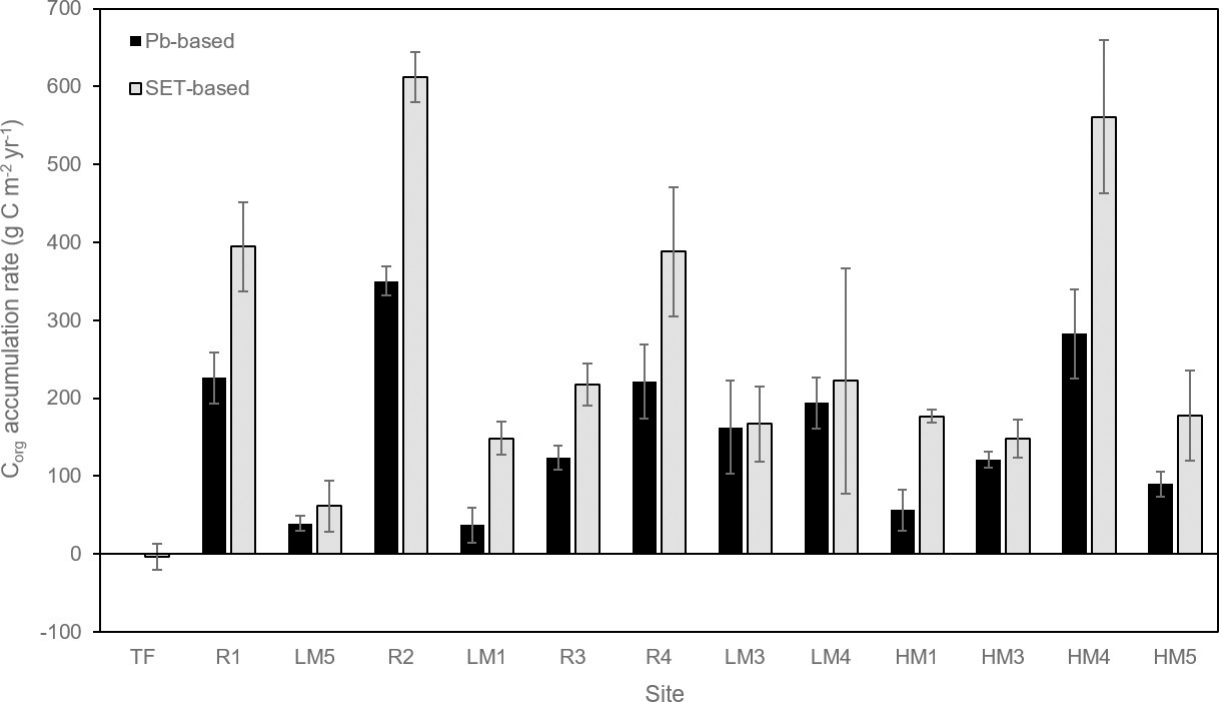
Carbon accumulation rates (±SE) from each site, with both ^210^Pb-based and SET-based rates shown. Sites are arranged in order of increasing elevation.

Carbon accumulation rates calculated with ^210^Pb-based accretion rates ranged from 0 g C m^-2^ yr^-1^ (TF) to 350.23 g C m^-2^ yr^-1^ (R2), averaging 123.00 g C m^-2^ yr^-1^ at natural marsh sites and 230.49 g C m^-2^ yr^-1^ at restored sites. Carbon accumulation rates calculated with SET-based elevation change rates ranged from -2.95 g C m^-2^ yr^-1^ (TF) to 611.69 g C m^-2^ yr^-1^ (R2), with an average of 207.82 g C m^-2^ yr^-1^ at natural marsh sites and 402.93 g C m^-2^ yr^-1^ at restored sites (Fig 5, Table 2). Although the ^210^Pb and SET-based rates represent different time periods of record (100 years and 4–7 years, respectively), the same 30-cm depth was used for mean carbon density in both cases.

### Environmental characteristics

Porewater salinity, as measured at the end of the growing season in mid-August, ranged from 6 ppt (at HM1 adjacent to Hat Slough) to 20 ppt (at several sites), and averaged 15.5 ppt within both natural marsh sites and restored sites (Table 1).

Site elevations ranged from 2.15 m MLLW (TF) to 3.34 m MLLW (HM5). Undisturbed low marsh sites ranged from 2.48 to 2.71 m MLLW, while high marsh sites were approximately 0.5 m higher, ranging from 3.04 to 3.34 m MLLW. Elevations within the restoration area fell within the range of undisturbed low marsh elevations, ranging from 2.40 to 2.85 m MLLW (Table 1).

### Drivers of plant biomass, carbon stocks, and carbon accumulation rates

Plant biomass was more closely related to elevation than to salinity. Elevation positively correlated with aboveground biomass (Spearman’s rank correlation, ρ = 0.86, *P* = 0.0002, *n* = 13) while salinity did not correlate with aboveground biomass (Spearman’s rank correlation, ρ = -0.27, *P* = 0.45, *n* = 10). Among the natural marsh sites, Kruskal-Wallis tests determined that both aboveground and belowground biomass were significantly greater at high marsh sites compared to low marsh sites (Aboveground: *H* = 5.33, 1 *df*, *P* = 0.02; Belowground: *H* = 5.33, 1 *df*, *P* = 0.02).

Sediment carbon stocks were significantly and positively correlated with elevation, aboveground biomass, and belowground biomass, but the strongest relationship was with elevation (Spearman’s rank correlation, ρ = 0.86, *P* = 0.0003, *n* = 13). Carbon accumulation rates, however, only correlated with accretion rates such that high accretion rates led to high carbon accumulation rates (Spearman’s rank correlation, ρ = 0.97, *P* < 0.001, *n* = 13). These two rates are not independent however, with carbon accumulation calculated as the product of accretion rate and carbon density. No significant relationship was detected between carbon accumulation rates (both SET-based and ^210^Pb-based) and elevation, biomass, or even sediment carbon stocks (which are based on carbon density).

## Discussion

### Plant biomass

Within natural marshes, aboveground biomass at high marsh sites was approximately three times greater than at low marsh sites. Belowground biomass showed even more of a contrast, with over four times greater biomass at high marsh sites. The restored sites varied considerably in terms of both aboveground and belowground biomass. Restored site R4 had a higher aboveground biomass than other low marsh sites. This was the southernmost site in the restored marsh where vegetation was dominated by cattails (*Typha latifolia* and *T*. *angustifolia*) and maritime bulrush (*Bolboschoenus maritimus*), both relatively productive species. Cattails were not found at any other site. Their prevalence in the restoration area is likely a result of their occurrence across the site’s freshwater wetlands prior to restoration, and their ability to persist after restoration despite suboptimal conditions [22]. Restored sites R1 and R2 contained very little aboveground biomass. These two sites were located near the northern dike breach where a developing sand splay reduced the area’s capacity for permanent vegetation establishment.

### Sediment characteristics

Sediment profiles at all four restored sites showed a spike in organic material between 15 and 20 cm in depth. The cause of this spike is unclear but it is hypothesized to be from either pre-restoration standing plant material that was quickly buried after the dike breach (or trampled as part of the levee removal process), or a layer of material originating outside the site that was deposited during a storm event. The latter is perhaps more likely since this layer looked more like small pieces of wood instead of dead herbaceous material. In any case, the depth of this layer reasonably corresponds to the depth of sediment we expect to have accumulated after the dike breach, based on SET elevation change rates from the last four years.

All other undisturbed sites (beyond the restoration area) generally exhibited typical sediment profile patterns with highest percent organic matter and carbon at the surface, and a decrease with depth due to organic material decomposition and diminishing root growth. Low marsh and tidal flat sites tended to have lower levels of carbon at the surface, but they still showed some decrease with depth. Bulk density was generally lowest at the surface as expected, increasing with depth due to the compaction and decomposition of less dense organic matter.

The restored marsh sites, although low in elevation, had a mean sediment bulk density comparable to that of the high marsh sites. Despite the restoration and natural marsh sites having a similar mean bulk density and percentage of fine-grained sediments, organic content, carbon content, and carbon density were all lower in the restoration area. In fact, these values were low in the restored sites even when compared to just the natural low marsh sites, which occur within the same elevation range (e.g., restored site carbon density averaged 0.015 g C cm^-3^ compared to 0.017 g C cm^-3^ in natural low marshes), Because the restoration area has experienced very rapid sediment deposition since the dike breaches in 2012, plant material has had little opportunity to develop and influence the sediment profile. However, even the sites with little or no plant material (TF, R1, and R2) still contained a small amount of sediment carbon, indicating that this carbon is likely allochthonous, presumably deposited with the incoming sediment independent of the *in situ* vegetation. Similarly, Wollenberg et al. [23] measured a 2.3% carbon content in newly deposited sediment in the Bay of Fundy, concluding that short-term soil carbon density was driven by the inherent carbon content of incoming sediment.

Sediment carbon density in the Stillaguamish River estuary is low relative to many other estuaries; indeed the average values here are lower than the United States mean of 0.027 g cm^-3^ [24]. Sediment organic matter content, carbon content, and carbon density across the Stillaguamish River estuary are also slightly lower than those reported for the neighboring Snohomish River estuary near Everett, WA [12]. For example, carbon density at six Snohomish tidal marsh sites ranged from 0.018 g C cm^-3^ to 0.032 g C cm^-3^, whereas Stillaguamish marsh carbon density ranged from 0.012 g C cm^-3^ to 0.026 g C cm^-3^. Oregon tidal marsh carbon density is higher than both these Washington estuaries, with an Oregon mean of 0.034 g C cm^-3^ [25] compared to this study’s natural marsh mean of 0.20 g C cm^-3^. Although these two neighboring estuaries are only separated by approximately 20 miles, the most likely explanation for the difference in sediment characteristics is their differing hydrodynamics. The Stillaguamish estuary marshes are located at the northern end of Port Susan Bay with full exposure to waves from winter storms. In contrast, the Snohomish estuary marshes are situated further inland, where they are more protected from storms, allowing finer sediments to settle. Having blue carbon data from both estuaries provides a window into the variability we might expect to see across the Salish Sea, in terms of both storm exposure and access to riverine sediments.

### Accretion and elevation change rates

All sites, with the exception of the tidal flat site, are accumulating sediments and gaining elevation at a rate that exceeds current and even some predicted future rates of sea level rise, indicating that the estuary is not currently sediment-limited.

SETs in Zones 1 and 3 have seen a fairly steady increase in elevation over their 7-year period of record. These zones are nearest to the Stillaguamish River mouth and riverine sediment supply. Previously at Zones 4 and 5 (farthest from the river), sediment starvation and wave-induced erosion were believed to be deteriorating the low marsh and causing the high marsh to retreat. However, these areas have seen very dynamic conditions in the most recent years, with pools and channels encroaching in one year, and an influx of large woody debris and sediment deposits in the next. Although these dynamic conditions lead to more intermittent deposition rather than steady and predictable accretion, they appear to be slowing erosion in these areas.

The Zone 2 restored site SETs have shown 2.74 cm yr^-1^ of elevation change on average, over their four-year period of record. This rate is nearly three times that of the other undisturbed marsh sites. While it was expected and desired that the return of tidal and riverine pulsing to this formerly hydrologically isolated and subsided farmland would result in enhanced sedimentation, the rates observed here are remarkably high. These rates are higher than the 0.5–0.8 cm yr^-1^ calculated by Nowacki and Grossman [26] based on their measured suspended sediment fluxes and our mean bulk density, suggesting that perhaps an assumption of uniform sediment distribution is not accurate. Despite our initial high rates of elevation change, it is expected that as infill continues and the elevation inside the restoration zone approaches that of the adjacent undisturbed marshes, rates of elevation change will gradually decline. These initial high rates demonstrate that the restored marsh has the capacity to restore its elevation without additional human intervention, and this is likely to apply to other Puget Sound estuaries with undammed mountainous watersheds.

We originally predicted that SET-based elevation change rates and ^210^Pb-based accretion rates would be comparable because they measure similar processes of sediment deposition and shallow subsidence. However, we found the ^210^Pb accretion rates to be consistently lower than SET rates, with an average ^210^Pb:SET ratio of 0.57 across the eight natural marsh sites. One possible explanation for this difference is that the ^210^Pb method does not measure the most recent depositional layer with as much precision as the SET method, providing only one data point over the top 2 cm. This may result in the most recently deposited sediment not being fully accounted for, which would reduce the reported rate. Additionally, this surface layer may be compacted somewhat during the coring process. Another possible reason for the discrepancy is that SETs have a shorter length of record. Very few studies have used both methods at the same sites to allow for a comparison, but Breithaupt et al. [27] also found that SETs recorded a higher rate of accretion than that measured by ^210^Pb. Our results indicate that the two methods cannot be used interchangeably in the Stillaguamish estuary, although one rate may potentially be predicted from the other with this site-specific ratio.

### Carbon stocks and accumulation rates

Global average rates of salt marsh carbon accumulation are generally higher than those reported here for Stillaguamish natural marshes. For example, Mcleod et al. [2] reported a global average rate of 218 g C m^-2^ yr^-1^. A more recent review by [28] reported a global average of 244.7 g C m^-2^ yr^-1^, but a lower NE Pacific regional average (based on eight California sites) of 173.6 g C m^-2^ yr^-1^. However, some of the studies included in those global reviews used shorter-term accretion methods such as feldspar, which overestimate long-term accretion. Other studies along the west coast of North America report natural marsh rates more comparable to ours (all using ^210^Pb dating), including an estimated 79 g C m^-2^ yr^-1^ from San Francisco Bay [29], 77 g C m^-2^ yr^-1^ from Oregon [25], and 115 g C m^-2^ yr^-1^ from British Columbia, Canada [30]. Within the Puget Sound, Crooks et al. [12] reported 110 g C m^-2^ yr^-1^ from a natural marsh in the Snohomish estuary and Drexler et al. [13] reported a 50-year rate of 118 g C m^-2^ yr^-1^ from the Nisqually River estuary, both similar to our Stillaguamish average of 123 g C m^-2^ yr^-1^.

Few studies report carbon sequestration rates following marsh restoration. In Oregon, Brophy et al. reported 80 g C m^-2^ yr^-1^ in Columbia River estuary restored sites [10] and 150 g C m^-2^ yr^-1^ in Tillamook Bay restored sites [11], both lower than Stillaguamish rates. Drexler et al. [13] reported 81 to 210 g C m^-2^ yr^-1^ from the Nisqually River estuary. Snohomish estuary rates, however, were most similar to Stillaguamish rates, with up to 352 g C m^-2^ yr^-1^ based on 1.61 cm yr^-1^ of accretion [12]. Of course, it is not fair to compare one restored site to another without accounting for differences in vertical accommodation space, which is affected by site subsidence prior to restoration and time since restoration, but such an analysis is beyond the scope of this study.

Carbon stocks and accumulation rates are driven by different processes in the Stillaguamish River estuary. In other words, a high carbon stock does not necessarily imply a high carbon accumulation rate. Carbon stocks were highest at high marsh sites, whereas carbon accumulation rates at those same sites were moderate. Stocks were most strongly related to elevation (as a proxy for tidal inundation frequency), whereas accumulation rates were only predicted by sediment accretion rates, similar to the findings of Wollenberg et al. [23] and Peck et al. [25]. Accretion rates themselves are quite variable throughout the Stillaguamish estuary, ranging from 0.24 to 1.24 cm yr^-1^ just within natural marsh sites. These rates are clearly driven not just by estuary-scale conditions but also by smaller-scale sediment dynamics that warrant further study.

When comparing restored sites to just the natural low marsh sites, we might expect those carbon stocks and accumulation rates to be more similar because those sites exist within the same elevation range. However the mean sediment carbon stock in restored sites (4.43 g C m^-2^) was still lower than in the natural low marsh sites (5.18 g C m^-2^), suggesting that sediment carbon density in the restored marsh is still in the process of recovering after restoration. Since the restored marsh has had such high accretion rates from mineral sediment inputs (1.57 cm yr^-1^, versus 0.58 cm yr^-1^ in natural low marshes), we expect it may be several more years before the restoration soil profile resembles the natural sites.

A few studies have reported carbon accumulation rates calculated with SET elevation change rates [e.g., 31–33] but most use radiometric (^210^Pb or ^137^Cs) accretion rates. We have reported both SET and ^210^Pb rates here to allow for comparisons with both types of studies. SET-based carbon accumulation rates here were all higher, and on average nearly twice the ^210^Pb-based carbon accumulation rates, so they cannot be considered interchangeable. Considering that most studies use ^210^Pb-based carbon accumulation rates, the rest of our discussion emphasizes those rates.

### Implications for carbon finance and restoration

Since the Stillaguamish River estuary restored marsh was 0.5 to 1.0 m below the elevation of adjacent natural marshes at the time of dike removal, the restored marsh is expected to continue accumulating sediment until it reaches its former elevation. Assuming a constant sediment carbon density (likely conservative since it is currently lower than adjacent marshes), the entire 60-hectare restored marsh is expected to accumulate approximately 4,500 to 9,000 Tonnes of carbon, which is equivalent to removing approximately 3,500 to 7,000 cars from the roads for one year. That amount of carbon is worth approximately $65,000 to $165,000 using a national average carbon offset price of $4 to $5 per Tonne CO_2_. Once its elevation reaches an equilibrium with the surrounding marsh, the restored marsh is expected to continue accumulating carbon at a rate similar to that of the surrounding marsh, estimated at 1.23 Tonnes C ha^-1^ yr^-1^, or approximately 75 Tonnes C yr^-1^ within the entire restoration site. This carbon accumulation rate is valued at approximately $20 ha^-1^ yr^-1^, or $1,200 yr^-1^ within the entire restored area.

The national carbon offset value is currently quite low compared to the estimated social cost of carbon of $41 per Tonne [4]. There is some indication that the carbon offset value awarded to blue carbon projects may increase, in consideration of the many other co-benefits that marsh restoration provides, including but not limited to coastal storm protection, biodiversity, tourism, and fisheries enhancement. Until then, carbon credits alone are unlikely to fund an entire restoration project. However, although the monetary value of carbon sequestration is currently low, there are other potential benefits to recognizing and quantifying the climate benefits of marsh restoration: (1) the climate benefits of restoration may garner interest from additional funders seeking to support a project with a climate change mitigation component; (2) annual carbon offset payments may help fund monitoring efforts, which are typically difficult to fund; and (3) blue carbon adds to a long list of other marsh restoration benefits, increasing justification for restoration.

Tidal wetland restoration projects tend to be relatively small, losing cost-effectiveness once the carbon quantification, feasibility, design, and monitoring are accounted for [e.g., 34]. Fortunately, it is possible to group smaller carbon finance projects together within a larger project area to increase cost-effectiveness. Larger project areas of at least 500 ha could be at the scale of an estuary or even a region, and the individual restorations need not be implemented at the same time. This possibility should be an important consideration for tidal wetland restoration projects.

### Recommended next steps

This study expands the amount of PNW blue carbon data available to scientists, managers, and policymakers. To our knowledge no other blue carbon study in the PNW has included carbon accumulation rates from both ^210^Pb and SET measurements, in addition to C stocks within all three marsh carbon pools (i.e., sediment, aboveground biomass, and belowground biomass). The extensive dataset generated by this study can contribute to a feasibility analysis that determines if the site would be appropriate for the carbon market, using the Port Susan Bay Preserve restored marsh as an example for other potential projects within the region. This study will also facilitate comparisons of carbon stocks and accumulation rates across estuaries, which until only recently were not possible due to a lack of data within the region.

Some further research is recommended that was outside the scope of this study, to allow for a full feasibility analysis of the estuary’s carbon finance potential. Greenhouse gas (CO_2_, CH_4_, and N_2_0) emissions must be quantified for a complete blue carbon assessment. Even with substantial carbon sequestration, the estuary’s brackish marshes could simultaneously be emitting greenhouse gases, and it is yet unknown whether the estuary is a net greenhouse gas sink or source. It is possible to use published emission data from other estuaries but published methane (CH_4_) emissions can be quite variable, particularly from brackish marshes such as those in the Stillaguamish estuary, therefore site-specific data are preferred.

Carbon offset credits are awarded based on net autochthonous carbon accumulation (carbon produced *in situ*). Allochthonous carbon (carbon produced outside the system) must be subtracted from the carbon balance for crediting purposes. Further study using stable isotopes would allow for a differentiation between autochthonous and allochthonous carbon to provide this information. We expect that the majority of carbon sequestered in the Stillaguamish estuary restored marsh is allochthonous, given that sediment carbon content is relatively low [35] and a similar restored marsh in Puget Sound reported high allochthonous carbon contribution based on stable isotopes [36].

While much of the sequestered carbon in the restored marsh may be imported from elsewhere, the marsh may also be exporting carbon from its aboveground plant production. The quantity and fate of exported carbon can be difficult to track [37] and it is currently unaccounted for in this study. However, exported carbon can be a substantial contributor to the carbon budget of the larger coastal ocean [38].

Both the Stillaguamish and Snohomish estuaries have demonstrated relatively high rates of accretion (and consequently carbon accumulation) immediately following restoration, higher than rates reported from Oregon estuaries [10, 11] and southern Puget Sound [13] but comparable to rates from the Columbia River estuary [39]. It would be helpful to expand this assessment to restored marshes in other PNW estuaries to determine whether this high capacity for accretion is estuary-specific or region-specific.

## Conclusion

There is a need for a better understanding of the tidal marsh response to restoration, to facilitate carbon finance mechanisms for restoration projects. For this study we report biomass and sediment carbon stocks, sediment accretion rates, elevation change rates, and carbon accumulation rates, and compare these results among natural marsh, restored marsh, and tidal flat sites across the Stillaguamish estuary, Washington, USA. We found that the restoration of tidal and riverine flooding with dike removal here has greatly enhanced rates of sediment accretion and carbon accumulation in the restored marsh above natural marsh rates, despite relatively low sediment carbon densities and stocks. Thus, we caution that high carbon stocks do not necessarily imply high carbon accumulation rates. Carbon stocks and accumulation rates varied independently of each other, with stocks driven primarily by elevation, and rates driven primarily by accretion rates.

This is also one of few studies to compare ^210^Pb accretion rates to SET rates of elevation change. SET rates here were nearly twice the corresponding ^210^Pb rates, indicating that the two methods cannot be used interchangeably without a conversion. Whether from ^210^Pb or SETs, all rates across the Stillaguamish marsh sites were higher than the local rate of sea level rise, indicating that restoration here has successfully allowed the marsh to recover from subsidence while simultaneously providing resilience to sea level rise and carbon accumulation.

## Supporting information

S1 Fig Relationship between Organic Matter (OM) and organic carbon (Corg) content, based on a subset of 124 sediment core samples. (TIF)

S2 Fig Relationship between the percentage of fine-grained particles and sediment Organic Matter (OM) content, based on a subset of 154 sediment core samples. (TIF)

## References

[1] Costanza R, de Groot R, Sutton P, van der Ploeg S, Anderson SJ, Kubiszewski I, et al. Changes in the global value of ecosystem services. Glob Environ Change. 2014;26:152–8.

[2] Mcleod E, Chmura GL, Bouillon S, Salm R, Bjork M, Duarte CM, et al. A blueprint for blue carbon: Toward an improved understanding of the role of vegetated coastal habitats in sequestering CO_2_. Front Ecol Environ. 2011;9(10):552–60.

[3] Nelleman C, Corcoran E, Duarte CM, Valdes L, DeYoung C, Fonseca L, et al. Blue carbon: A rapid response assessment. Report prepared for the United Nations Environment Programme, Arendal, Norway; 2009.

[4] Pendleton L, Donato DC, Murray BC, Crooks S, Jenkins WA, Sifleet S, et al. Estimating global “blue carbon” emissions from conversion and degradation of vegetated coastal ecosystems. PLoS One. 2012;7:e43542. doi: 10.1371/journal.pone.0043542 22962585 PMC3433453

[5] Spivak AC, Sanderman J, Bowen JL, Canuel EA, Hopkinson CS. Global-change controls on soil-carbon accumulation and loss in coastal vegetated ecosystems. Nat Geosci. 2019;12:685–92.

[6] Brophy LS, Greene CM, Hare VC, Holycross B, Lanier A, Heady WN, et al. Insights into estuary habitat loss in the western United States using a new method for mapping maximum extend of tidal wetlands. PLoS ONE. 2019;14(8):e0218558. doi: 10.1371/journal.pone.0218558 31412030 PMC6693690

[7] Bayraktarov E, Saunders MI, Abdullah S, Mills M, Beher J, Possingham HP, et al. The cost and feasibility of marine coastal restoration. Ecol Appl. 2016;26:1055–74. doi: 10.1890/15-1077 27509748

[8] Wylie L, Sutton-Grier AE, Moore A. Keys to successful blue carbon projects: Lessons learned from global case studies. Mar Policy. 2016;65:76–84.

[9] Troxler TG, Kennedy HA, Crooks S, Sutton-Grier AE. Introduction of coastal wetlands into the IPCC greenhouse gas inventory methodological guidance. In: Windham-Myers L, Crooks S, Troxler T, editors. A blue carbon primer: The state of coastal wetland carbon science, practice, and policy. Florida: CRC Press; 2019. p. 217–34.

[10] Brophy LS, Brown LA, Ewald MJ, Peck EK. Baseline monitoring at Walloskee-Youngs restoration site, 2015, Part 2: Blue carbon, ecosystem drivers and biotic responses. Report prepared for U.S. Fish and Wildlife Service, Portland, Oregon; 2017.

[11] Brophy LS, Peck EK, Bailey SJ, Cornu CE, Wheatcroft RA, Brown LA, et al. Southern Flow Corridor effectiveness monitoring, 2015–2017: Sediment accretion and blue carbon. Report prepared for Tillamook County and Tillamook Estuaries Partnership, Tillamook, Oregon; 2018.

[12] Crooks S, Rybczyk J, O’Connell K, Devier DL, Poppe K, Emmett-Mattox S. Coastal blue carbon opportunity assessment for the Snohomish Estuary: The climate benefits of estuary restoration. Report prepared for Restore America’s Estuaries, Arlington, Virginia; 2014.

[13] Drexler JZ, Woo I, Fuller CC, Nakai G. Carbon accumulation and vertical accretion in a restored versus historic salt marsh in southern Puget Sound, Washington, United States. Restor Ecol. 2019;27(5):1117–27.

[14] NOAA Tides and Currents: Sea Level Trends [Internet]. National Oceanic and Atmospheric Administration; c2021 [cited 2021 Feb 8]. Available from: https://tidesandcurrents.noaa.gov/sltrends/sltrends_station.shtml?id=9444900

[15] Lynch JC, Hensel P, Cahoon DR. The surface elevation table and marker horizon technique: A protocol for monitoring wetland elevation dynamics. Fort Collins (CO): National Park Service (US); 2015. Report No.: NPS/NCBN/NRR—2015/1078.

[16] Westlake D F. Comparisons of plant productivity. Biol Rev. 1963;38:385–425.

[17] Craft CB, Seneca ED, Broome SW. Loss on ignition and kjeldahl digestion for estimating organic carbon and total nitrogen in estuarine marsh soils: Calibration with dry combustion. Estuaries Coast. 1991;14(2):175–9.

[18] Robbins JA, Edgington DN, Kemp ALW. Comparative 210Pb, 137Cs and pollen chronologies of sediments from Lake Ontario and Erie. Quat Res. 1978;10(2):256–78.

[19] R Core Team. R: A language and environment for statistical computing [software]. R Foundation for Statistical Computing. 2018. Available from: https://www.R-project.org

[20] Foster IDL, Mighall TM, Proffitt H, Walling DE, Owens PN. Post-depositional ^137^Cs mobility in the sediments of three shallow coastal lagoons, SW England. J Paleolimnol. 2006;35:881–95.

[21] Drexler JZ, Fuller CC, Archfield S. The approaching obsolescence of 137Cs dating of wetland soils in North America. Quaternary Sci Rev. 2018;199:83–96.

[22] Fuller R. Stillaguamish Estuary monitoring report: Summary of current conditions and trends. Report prepared for The Nature Conservancy of Washington, Seattle, WA; 2017.

[23] Wollenberg JT, Ollerhead J, Chmura GL. Rapid carbon accumulation following managed realignment on the Bay of Fundy. PLoS One. 2018;13:e0193930. doi: 10.1371/journal.pone.0193930 29561874 PMC5862474

[24] Holmquist JR, Windham-Myers L, Bliss N, Crooks S, Morris JT, Megnigal JP, et al. Accuracy and precision of tidal wetland soil carbon mapping in the conterminous United States. Sci Rep. 2018;8:9478. doi: 10.1038/s41598-018-26948-7 29930337 PMC6013439

[25] Peck EK, Wheatcroft RA, Brophy LS. Controls on sediment accretion and blue carbon burial in tidal saline wetlands: Insights from the Oregon coast, USA. J Geophys Res Biogeosci. 2020;125(2): e2019JG005464.

[26] Nowacki DJ, Grossman EE. Sediment transport in a restored, river-influenced Pacific Northwest estuary. Estuar Coast Shelf S. 2020;242:106869.

[27] Breithaupt JF, Smoak JM, Byrne RH, Waters MN, Moyer MP, Sanders CJ. Avoiding timescale bias in assessments of coastal wetland vertical change. Limnol Oceanogr. 2018;63:S477–95. doi: 10.1002/lno.10783 29937578 PMC5993342

[28] Ouyang X, Lee SY. Updated estimates of carbon accumulation rates in coastal marsh sediments. Biogeosciences. 2014;11:5057–71.

[29] Callaway JC, Borgnis EL, Turner RE, Milan CS. Carbon sequestration and sediment accretion in San Francisco Bay tidal wetlands. Estuaries Coast. 2012;35:1163–81.

[30] Chastain S, Kohfeld KE, Pellatt MG, Garcia CO, Gailis M. Carbon stocks and accumulation rates in salt marshes of the Pacific coast of Canada. Manuscript in preparation.

[31] Howe AJ, Rodriguez JF, Saco PM. Surface evolution and carbon sequestration in disturbed and undisturbed wetland soils of the Hunter estuary, southeast Australia. Estuar Coast Shelf S. 2009;84:75–83.

[32] Lovelock CE, Adame MF, Bennion V, Hayes M, O’Mara J, Reef R, et al. Contemporary rates of carbon sequestration through vertical accretion of sediments in mangrove forests and saltmarshes of South East Queensland, Australia. Estuaries Coast. 2014;37:763–71.

[33] Rogers K, Saintilan N, Copeland C. Managed retreat of saline coastal wetlands: Challenges and opportunities. Estuaries Coast. 2013;37:67–78.

[34] Crooks S, Beers L, Settelmyer S, Swails E, Emmett-Mattox S, Cornu C. Scoping assessment for Pacific Northwest blue carbon finance projects. Report prepared by Silvestrum Climate Associates, TerraCarbon LLC, Strategic Collaborations LLC, and the Institute for Applied Ecology; 2020.

[35] Needelman BA, Emmer IM, Emmett-Mattox S, Crooks S, Megonigal JP, Myers D, et al. The science and policy of the verified carbon standard methodology for tidal wetland and seagrass restoration. Estuaries Coast. 2018;41:2159–71.

[36] Drexler JZ, Davis MJ, Woo I, De La Cruz S. Carbon sources in the sediments of a restoring vs. historically unaltered salt marsh. Estuaries Coast. 2020;43:1345–60.

[37] Bauer J, Wei-Jun C, Raymond PA, Bianchi TS, Hopkinson CS, Regnier PAG. The changing carbon cycle of the coastal ocean. Nature. 2013;504:61–70. doi: 10.1038/nature12857 24305149

[38] Wang ZA, Kroeger KD, Ganju NK, Gonneea ME, Chu SN. Intertidal salt marshes as an important source of inorganic carbon to the coastal ocean. Limnol Oceanogr. 2016;61(5):1916–31.

[39] Diefenderfer HL, Coleman AM, Borde AB, Sinks IA. Hydraulic geometry and microtopography of tidal freshwater forested wetlands and implications for restoration, Columbia River, U.S.A. Ecohydrol Hydrobiol. 2008;8(2):339–361.

