# Supplementary figures and images for: Tidal marsh restoration enhances sediment accretion and carbon accumulation in the Stillaguamish River estuary, Washington
Source: PLoS One. 2021 Sep 10;16(9):e0257244. doi: 10.1371/journal.pone.0257244 (PMC8432862; doi:10.1371/journal.pone.0257244)

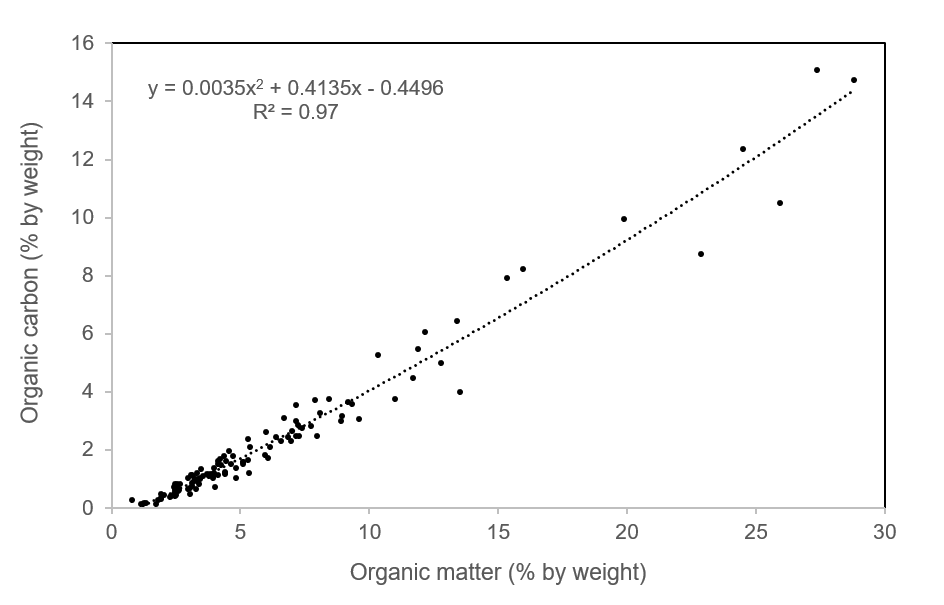

Supplement: S1 Fig — (TIF) [file pone.0257244.s001.tif]

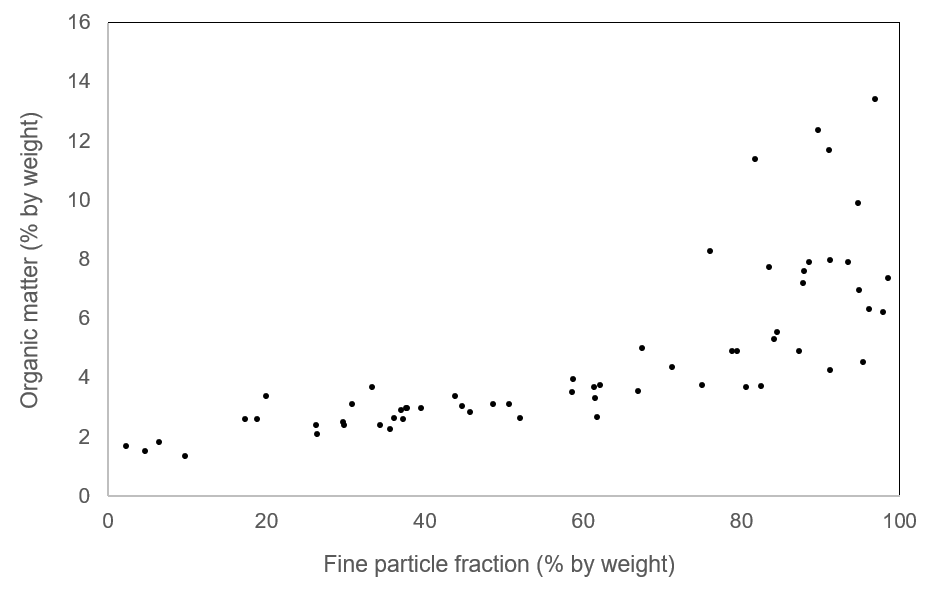

Supplement: S2 Fig — (TIF) [file pone.0257244.s002.tif]
